# RANGE OF MOTION AFTER BONE BLOCK PROCEDURES FOR SHOULDER INSTABILITY: SYSTEMATIC REVIEW

**DOI:** 10.1590/1413-785220243201e273366

**Published:** 2024-05-06

**Authors:** Paulo Henrique Schmidt Lara, Leandro Masini Ribeiro, Carlos Vicente Andreoli, Alberto de Castro Pochini, Paulo Santoro Belangero, Benno Ejnisman

**Affiliations:** 1. Federal University of São Paulo, São Paulo School of Medicine, Center of Sport Traumatology, São Paulo, SP, Brazil.

**Keywords:** Shoulder, Systematic Review, Orthopedic Surgery, Ombro, Revisão Sistemática, Cirurgia Ortopédica

## Abstract

**Objective:**

to determine the surgical indications for glenoid bone grafting associated with better postoperative ranges of motion.

**Methods:**

This systematic review was conducted according to PRISMA. The included studies were subdivided according to the criteria used to indicate glenoid bone graft surgery: group for radiological indications only (Group R), group for radiological indications associated with clinical indications (Group R + C), and group for arthroscopic indications (Group A). The extracted and evaluated data were the range of motion of the shoulder.

**Results:**

in the electronic search conducted in October 2022, 1567 articles were selected. After applying the inclusion criteria, 14 articles were selected for the systematic review. Regarding the ranges of motion, group A had the highest number of statistically positive results together with group R. Group A showed positive results in elevation parameters, loss of lateral rotation in adduction, and medial rotation in abduction. Group R showed positive results in lateral rotation in adduction and loss of lateral rotation in adduction. On the other hand, Group R + C was the one that presented the highest number of statistically negative results, in the following parameters: elevation, lateral rotation in abduction, loss of lateral rotation in adduction, and medial rotation in abduction.

**Conclusion:**

the subgroups presented variable results in the evaluated parameters; however, the groups with arthroscopic and radiological indications showed the highest number of positive results, with the latter group showing the best results regarding lateral rotation. ***Level of Evidence II, Systematic Reviews.***

## RESUMO

**Objetivo:**

*Determinar as indicações cirúrgicas de enxertia óssea da glenoide associadas aos melhores arcos de movimento no pós-operatório.*

**Métodos:**

*De acordo com o Preferred Reporting Items for Systematic Reviews and Meta-Analyses (PRISMA), 14 artigos de um total de 1.567, selecionados em busca eletrônica, foram escolhidos para a revisão sistemática. Os estudos incluídos foram subdivididos de acordo com os critérios de indicação da cirurgia: indicações somente radiológicas (grupo R), indicações radiológicas associadas a indicações clínicas (grupo R + C) e indicações artroscópicas (grupo A). Os dados avaliados foram os arcos de movimento do ombro.*

**Resultados:**

*Em relação aos arcos de movimento, os grupos que apresentaram a maior quantidade de resultados estatisticamente positivos foram o A – parâmetros elevação, perda de rotação lateral em adução e rotação medial em abdução – e o R – parâmetros rotação lateral em adução e perda de rotação lateral em adução. O grupo R + C apresentou a maior quantidade de resultados estatisticamente negativos nos parâmetros elevação, rotação lateral em abdução, perda de rotação lateral em adução e rotação medial em abdução.*

**Conclusão:**

*Os grupos de indicações artroscópicas e radiológicas apresentaram a maior quantidade de resultados positivos, sendo que o último apresentou os melhores resultados em relação à rotação lateral.* ***Nível de Evidência II, Revisão Sistemática.***

## INTRODUCTION

 Anterior shoulder dislocation is a complication to the evolution of recurrent shoulder instability and occurs in up to 60% of patients. ^1^^,^^2^ Determining the best surgeries for anterior shoulder instability is controversial, with several procedures created over time. According to studies, Bankart surgery, also known as anatomical repair, is the initial procedure in cases of anterior shoulder instability, being chosen in more than 90% of cases. ^3^^,^^4^

 The popularity of open Bankart repair has led to the development of the efficient arthroscopic Bankart repair, which has a recurrence rate of 6% and a review rate of 4.7% according to a systematic review. ^5^ Nevertheless, Burkhart and De Beer ^6^ have shown that the recurrence rate of instability was 67% in patients with large bone lesions (Bankart or Hill-Sachs) who underwent Bankart surgery and 89% in contact athletes with the same disease. This suggests that the effectiveness of Bankart surgery may be limited in the presence of bone lesions. 

 Consequently, the number of indications for glenoid bone graft surgery has increased. Initial studies of this type of surgery demonstrated a recurrence rate of 10% and surgical review rate of 14% in the Latarjet technique, ^7^^-^^9^ with some institutions abandoning this procedure. ^10^ However, recent studies have shown better success rates. A systematic review by Griesser et al. ^11^ has shown a recurrence rate of 2.9% and a subluxation rate of 5.8%. Specifically, in patients with bone lesions, the Latarjet technique had a recurrence rate of 4.7%, demonstrating superiority over anatomical surgery. ^12^ Nevertheless, the Latarjet technique is associated with a high rate of complications, occurring in up to 30% of cases. ^11^

In previous studies, glenoid bone grafting surgeries have shown lower recurrence rates and good functional results, making them frequently indicated. However, they are associated with complications such as neurological injuries and development of shoulder arthrosis. Thus, this systematic review mainly aimed to determine which indications for glenoid bone grafting surgeries are associated with better results in relation to arcs of movement in the postoperative period, helping in the appropriate choice of this modality of surgery.

Previous systematic reviews evaluated different aspects of glenoid bone grafting procedures. However, to our knowledge, no systematic review sought to determine which surgical indications would lead to better arcs of movement after surgery. With this, we seek to analyze the literature qualitatively and quantitatively to determine these indications.

## MATERIALS AND METHODS

### Search strategy in the literature

This systematic review was officially registered in PROSPERO on October 23, 2020 (CRD42020210462). This systematic review was conducted according to the guidelines of the International Preferred Reporting Items for Systematic Reviews and Meta-Analyses (PRISMA).

Electronic searches were performed using the Cochrane Library, PubMed, EMBASE, and LILACS databases in October 2022. Data from these databases were searched following the recommendations of Cochrane Collaboration, PRISMA, and Meta-analysis of Observational Studies in Epidemiology. To achieve maximum sensitivity in the search strategy, the terms “Latarjet” OR “Bristow” OR “Eden-Hybinette” OR “Bone block procedures” AND “Shoulder instability” were combined as keywords or MeSH terms. The reference list of all articles was reviewed for further identification of potentially relevant studies. The studies were evaluated using inclusion and exclusion criteria. There was no time limit on publications. There was no restriction regarding the language of publication. (Appendix 1)

### Selection criteria

Inclusion criteria were as follows: (1) randomized controlled trials (glenoid bone graft surgery vs. anatomical surgery or glenoid bone graft surgery vs glenoid bone graft surgery; (2) prospective cohort studies in which glenoid bone graft surgery was evaluated. The exclusion criteria were as follows: (1) retrospective studies, (2) case reports (less than five cases), and (3) studies in which the inclusion criteria of patients did not consider radiological criteria, radiological criteria associated with clinical criteria, or arthroscopic criteria.

### Data extraction and analysis

 Relevant information regarding the characteristics of the studies, evaluation of the methodological quality of the studies, measurements of the ranges of motion, and follow-up time were collected independently by two authors using a standard form. The Downs and Black ^13^ checklist and the Cochrane risk of bias tool for randomized trials ^14^^-^^17^ were used to assess the quality of cohort studies and randomized controlled trials, respectively. The Downs and Black checklist ^13^ ranges from 0-28 points, with a score of 26-28 considered excellent, 20-25 good, 15-19 regular, and lower than 15 bad. Interobserver agreement (3 authors) was evaluated using the kappa test. 

The studies were subdivided according to the main criterion used to indicate glenoid bone graft surgery:

- Radiological indication group (R) (10-25% anterior glenoid wear and/or off-track injuries)
- Radiological indication group associated with clinical indication (R + C) (same indications as radiological indication group + contact sports and/or ISIS (instability severity index score)) ≥ 4
- Arthroscopic indication group (A) (Hill-Sachs lesion with engagement)

The outcomes extracted and evaluated were: ranges of shoulder motion (elevation, loss of elevation, abduction, loss of abduction, lateral rotation in adduction, loss of lateral rotation in adduction, lateral rotation in abduction, loss of lateral rotation in abduction, medial rotation in abduction, loss of medial rotation in abduction, medial rotation in adduction, and loss of medial rotation in adduction).

### Statistical analysis

The significance level was set at 0.05 (5%). A complete descriptive analysis of the quantitative data was performed using mean, median, standard deviation, coefficient of variation, and confidence interval. The Z test was used to compare the groups in the evaluated parameters. Due to the qualitative characteristics of this systematic review, it was not possible to perform a meta-analysis. The agreement between the three authors for the Downs & Black checklist was measured using Fleiss’ kappa test for simultaneous analysis and Cohen’s kappa test for paired analysis.

## RESULTS

### Search results and quality of studies

**Figure 1.**
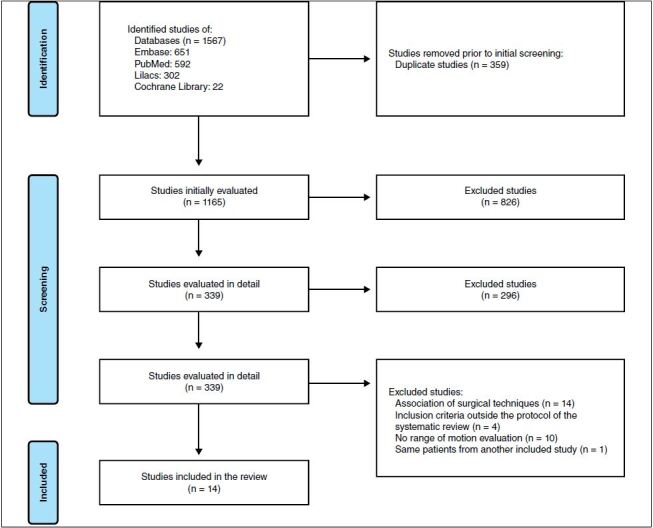
Flowchart based on the *International Preferred Reporting Items for Systematic Reviews and Meta-Analyses* (PRISMA).

 In the electronic search conducted in October 2022, 1567 articles were identified. After applying the inclusion criteria, 43 articles were selected and 29 articles were excluded (14 due to the association of surgical techniques, four due to the use of non-standardized inclusion criteria, 10 due to the absence of evaluation of the range of motion, and one due to the use of the same patients from another study already included). With this, a total of 14 articles were selected for the systematic review, which included 12 prospective cohort studies ^12^^,^^18^^-^^27^ and two randomized controlled trials. ^28^^,^^29^ A flow diagram based on PRISMA is shown in Figure 1 . In addition, the characteristics of the included studies and their methodological quality are presented in Table 1 . 

 Of the 12 included prospective cohort studies that were evaluated by the Downs & Black checklist, ^13^ seven (58.33%) were classified as weak, three (25%) as regular, and two (16.66%) as good. Regarding the agreement between authors, the Fleiss kappa test of the three authors showed a value of 0.842, which was classified as excellent. Appendix 3 shows the full results. 

### Demographic data

In the total of the studies, 659 shoulders were included, of which 548 (83.15%) were of men, 69 (10.47%) of women and 42 (6.37%) had no defined gender in the study. The mean follow-up was 43.01 months (23.7-90.0 months). It was not possible to calculate the mean age, since this type of data was not provided in all studies.

### Indications

 Four studies included ^20^^,^^21^^,^^27^^,^^28^ used only radiological criteria and contained a total of 206 shoulders. Five studies included ^18^^,^^19^^,^^24^^,^^25^^,^^29^ used clinical and radiological criteria, with 245 shoulders. Four studies included ^12^^,^^22^^,^^23^^,^^25^ used arthroscopic criteria, with a total of 208 shoulders. 

### Surgical techniques

Different surgical techniques were described in the articles selected for this systematic review and were performed according to the surgeons’ preferences and experiences. The techniques were used in the following frequencies: open Latarjet in 411 (66.91%) shoulders; arthroscopic Latarjet in 123 (18.66%) shoulders; open distal tibia graft in 50 (7.58%) shoulders; open Eden–Hybinette in 46 (6.98%) shoulders; and open Bristow in 29 (4.40%) shoulders.

### Ranges of motion

The outcomes extracted and evaluated were: ranges of shoulder motion (elevation, loss of elevation, abduction, loss of abduction, lateral rotation in adduction, loss of lateral rotation in adduction, lateral rotation in abduction, loss of lateral rotation in abduction, medial rotation in abduction, loss of medial rotation in abduction, medial rotation in adduction, and loss of medial rotation in adduction).

 Elevation was assessed in nine studies. ^12^^,^^21^^-^^27^^,^^29^ Elevation loss was evaluated in three studies. ^19^^,^^20^^,^^30^ Abduction was evaluated in two studies. ^24^^,^^25^ Abduction loss was evaluated in two studies. ^19^^,^^20^ Lateral rotation in adduction was evaluated in nine studies. ^12^^,^^18^^,^^21^^-^^23^^,^^25^^-^^27^^,^^29^ Loss of lateral rotation in adduction was evaluated in four studies. ^20^^,^^22^^,^^28^^,^^30^ Lateral rotation in abduction was evaluated in three studies. ^21^^,^^22^^,^^26^ Loss of lateral rotation in abduction was evaluated in four studies. ^19^^,^^20^^,^^22^^,^^30^ Medial rotation in abduction was evaluated in three studies. ^21^^,^^25^^,^^26^ Loss of medial rotation in abduction was evaluated in one study. ^19^ Medial rotation in adduction was evaluated in one study. ^27^

 Loss of medial rotation in adduction was evaluated in four studies. ^20^^,^^23^^,^^27^^,^^30^

**Table 1.** Characteristics of studies.

**Author**	**Type of study**	**Shoulders (n)**	**Surgical technique**	**Surgical indications**	**Range of motion**	**Quality of studies**	**Follow-up (months)**
Abdelhady et al 2015 ^18^	Prospective cohort	13 (10 men/3 women)	Latarjet open	1) Hill-Sachs < 20% humeral head diameter; 2) Ligament hyperlaxity	1) Lateral rotation in adduction	Weak	33.64
Abouelsoud and Abdelrahman 2015 ^28^	Randomized controlled trial	32 (no gender was mentioned)	Latarjet open (16) x Remplissage (16)	1) 3 episodes of dislocation in 12 months of conservative treatment; 2) Hill-Sachs 20-30% of the size of the humeral head on NMR	1) Loss of lateral rotation in adduction	Appendix 2	31.31
Ali et al. 2020 ^19^	Prospective cohort	48 (open Latarjet: 12 Men/3 women; arthrsoscopic Latarjet: 29 men/4 women)	Open Latarjet (15) x Arthroscopic Latarjet (33)	1) > 18 years old; 2) Osteochondral glenoid defect > 13.5%; 3) ISIS > 3 combined to seizure in the intermediate range of motion	Loss of: 1) Elevation; 2) Abduction; 3) Lateral rotation in abduction; 4) Medial rotation in abduction	Weak	30.5
Auffarth et al. 2008 ^20^	Prospective cohort	46 (40 men/6 women)	Open Eden-Hybinette	1) Glenoid defect > 5mm in length on AP and axial radiographs	Loss of: 1) Elevation; 2) Abduction; 3) Lateral rotation in adduction; 4) Lateral rotation in abduction	Weak	90
Belangero et al. 2021 ^29^	Randomized controlled trial	41 (37 men/4 women)	Open Latarjet (22) x Open Bristow (19)	1) Competitive sport 2) 10-20% anterior glenoid wear (CT)	1) Elevation; 2) Lateral rotation in adduction	Appendix 2	60
Burkhart et al. 2007 ^12^	Prospective cohort	47 (46 men/1 woman)	Open Latarjet	1) Inverted pear glenoid; 2) Hill-Sachs with engaging	1) Elevation; 2) Lateral rotation in adduction	Weak	52
Cautiero et al. 2017 ^30^	Prospective cohort	26 (does not mention genders)	Open Latarjet	1) Glenoid bone loss > 15% (CT - PICO method); 2) Hill-Sachs > 1/3 humeral head diameter; 3) Competitive sport of contact or above the head; 4) HAGL injury; 5) Very thin capsular tissue	Loss of: 1) Elevation; 2) Lateral rotation in adduction; 3) Medial rotation in adduction	Weak	53
Frank et al. 2018 ^21^	Prospective cohort	100 (96 men/4 women)	Open Latarjet (50) x Open tibia allograft (50)	1) Glenoid bone loss > 15%; 2) Tibia allograft preference: glenoid bone loss > 25%; important cartilaginous component	1) Elevation; 2) Medial rotation in abduction; 3) Lateral rotation in abduction; 4) Extension; 5) Abduction	Regular	45
Kordasiewicz et al 2016 ^22^	Prospective cohort	48 (46 men/2 women)	Open Latarjet (48) x Arthroscopic Latarjet (62)	1) Hill-Sachs engaging injury	1) Elevation; 2) Abduction; 3) Lateral rotation in adduction; 4) Lateral rotation in abduction	Regular	54.2
Kordasiewicz et al. 2019 ^23^	Prospective cohort	90 (80 men/10 women)	Latarjet Arthroscopic	1) Hill-Sachs engaging injury	1) Elevation; 2) Abduction; 3) Lateral rotation in adduction; 4) Lateral rotation in abduction	Regular	23.7
Moroder et al. 2018 ^24^	Prospective cohort	25 (13 men/12 women)	Open Latarjet (15) x Open Bristow (10)	1) > 40 years old; 2) Glenoid defect associated with clinically compensated cuff injuries	1) Elevation; 2) Abduction;	Weak	29
Vadalà et al. 2017 ^25^	Prospective cohort	24 (22 men/2 women)	Open Latarjet	1) ISIS > 6; 2) Participation in sports	1) Elevation; 2) Abduction; 3) Lateral rotation in adduction; 4) Lateral rotation in abduction	Weak	24
Yang et al. 2018 ^26^	Prospective cohort	91 (86 men/5 women)	Open Latarjet	Hill-Sachs injury with engagement	1) Elevation; 2) Lateral rotation in adduction; 3) Lateral rotation in abduction; 4) Medial rotation in abduction	Good	38.4
Zhu et al. 2017 ^27^	Prospective cohort	44 (32 men/12 women)	Open Latarjet	1) Glenoid bone loss > 20%	1) Elevation; 2) Lateral rotation in adduction; 3) Medial rotation in adduction	Weak	37.4

NMR: nuclear magnetic resonance; CT: computed tomography.

### Comparisons between the evaluated groups

Ranges of motion

The following parameters were evaluated

a. Elevation

This parameter was evaluated in all groups. The best results were found in Group A, with a statistically significant difference compared to the other groups.

b. Loss of elevation

This parameter was not evaluated in all groups, not allowing a comparison between them.

c. Abduction

This parameter was not evaluated in all groups, not allowing a comparison between them.

d. Loss of abduction

This parameter was not evaluated in all groups, not allowing a comparison between them.

e. Lateral rotation in adduction (LR 1)

This parameter was evaluated in all groups. The best results were found in groups R and R + C, with a statistically significant difference compared to Group A. Complete results can be seen in Table 2 .

f. Loss of lateral rotation in adduction (LLR 1)

This parameter was evaluated in all groups. Groups R and A presented better results, with statistical significance (p < 0.001). Complete results can be seen in Table 2 .

g. Lateral rotation in abduction (LR 2)

This parameter was evaluated in all groups. Group R presented better results, with statistical significance compared to the other groups. Complete results can be seen in Table 2 .

h. Loss of lateral rotation in abduction

This parameter was not evaluated in all groups, not allowing a comparison between them.

i. Medial rotation in abduction

This parameter was evaluated in all groups. The best results were found in Group R + C, with a statistically significant difference compared to the other groups (p < 0.001).

j. Loss of medial rotation in abduction

This parameter was not evaluated in all groups, not allowing a comparison between them.

k. Medial rotation in adduction

This parameter was not evaluated in all groups, not allowing a comparison between them.

l. Loss of medial rotation in adduction

This parameter was not evaluated in all groups, not allowing a comparison between them.

 Briefly, there were the following results of the groups regarding the ranges of motion that are shown in Chart 1 . 

**Table 2.** Ranges of motion involving lateral rotation.

			**Mean**	**SD**	**N**
LR 1 Group R + C Group A		Group R	68.9	13	190
		68.4	7	79	
		53.7	17.3	275	
			**Grp R**	**Grp R + C**	
LR1	Grp R + C		0.654		
	Grp A		< 0.001	< 0.001	

			**Mean**	**SD**	**N**
LLR 1 Group R + C Group A		Group R	7.1	3	62
		13	5	26	
		7	5	47	
			**Grp R**	**Grp R + C**	
LLR1	Grp R + C		< 0.001		
	Grp A		0.903	< 0.001	

			**Mean**	**SD**	**N**
LR 2 Group R + C Group A		Group R	82	12.4	100
		62.9	13	79	
		66.7	16.4	138	
			**Grp R**	**Grp R + C**	
LR2	Grp R + C		< 0.001		
	Grp A		< 0.001	< 0.001	

LR 1: lateral rotation in adduction; LLR 1: loss of lateral rotation in adduction; LR 2: lateral rotation in abduction.

**Chart 1.** Summary of results regarding ranges of motion.

	**Statistically positive results**	**Statistically negative results**
Elevation	Group A	Group R Group R + C
Lateral rotation in adduction	Group R Group R + C	Group A
Lateral rotation in abduction	Group R	Group R + C Group A
Loss of lateral rotation in adduction	Group R Group A	Group R + C
Medial rotation in abduction	Group A	Group R Group R + C

## DISCUSSION

In this systematic review, 23 studies were included, totaling 659 shoulders. Only prospective studies in which the indications for the choice of glenoid bone graft surgery were explicitly described were chosen to avoid the selection bias that can occur in retrospective studies. However, the analysis of the included studies showed a low methodological quality. Thus, the indications for the choice of glenoid bone graft surgery are very variable in the literature and are controversial. This systematic review aimed to determine which surgical indications would lead to better results regarding ranges of motion. For this, we divided the indications into three groups: radiological, clinical and radiological, and arthroscopic indications.

Among the subgroups of indications included in this systematic review, the largest number of shoulders that underwent the glenoid bone grafting procedure was in the group of radiological and clinical indications (245 shoulders). In general, variable results were observed, with no group presenting better results for all variables studied.

 In the radiological indications group (Group R), the indications were: 10-25% anterior glenoid wear and/or off-track injury. According to Burkhart and De Beer ^6^ , glenoid bone loss is a significant risk factor for recurrence of instability after Bankart repair. Initially, it was believed that the critical amount of glenoid bone loss was 25%. ^6^^,^^31^ Nevertheless, a recent cadaver study suggested that a 20% loss decreases shoulder stability after Bankart repair. ^32^ Yamamoto et al. ^33^ conducted a study to evaluate what would be the subcritical bone loss of the glenoid and found a value between 17-25%. 

 As described by Di Giacomo, Itoi, and Burkhart, ^34^ it is important to assess both glenoid and humeral bone loss and the relationship between them, as well as glenoid track measurements. Recent biomechanical studies on bipolar bone loss and the glenoid track concept have revealed a significant decrease in shoulder stability, with glenoid defects as small as 10-15%. ^35^

 In Group R + C, studies were included in which the indications were the same as in Group R, associated with the practice of contact sports and/or ISIS ≥ 4. The score takes into account clinical and radiological criteria. Initially, a score from 6 indicated glenoid bone graft surgery; in scores above this value, a failure rate of 70% was reported in cases where anatomical repair was chosen. ^36^ It is worth noting that this score uses radiographs for indication, and in our study, only three studies used radiographs to decide which surgery to perform. Currently, the glenoid track instability management score (GTIMS) ^37^ was created, incorporating the concept of glenoid track to ISIS and using only tomography as radiological parameter and not radiographs. Patients with on-track lesions score 0 and off-track score 4 points. The other parameters are equal to ISIS and a score from 4 points indicates glenoid bone graft surgery. It is important to emphasize that in the GTMIS the presence of an off-track lesion already leads to a score of 4 points, indicating glenoid bone graft surgery, without the need for evaluation of other parameters. 

In Group A, the main indication was the presence of a Hill-Sachs lesion with engagement in the arthroscopic evaluation. We consider this mode of indication valid because it allows the evaluation of associated lesions, but with the anesthetized patient there may be an overindication of the glenoid bone grafting procedure because the patient is more relaxed by anesthesia. Due to this fact, we believe that the indication of glenoid bone graft surgery should be made in advance based on radiological and clinical data. This group of patients presented variable results in the parameters evaluated; however, it presented, along with the group with radiological indications, the highest number of statistically positive results. One hypothesis for these findings is of an overindication, leading to patients who did not need glenoid bone graft surgery being subjected to this type of surgery.

Concerning ranges of motion, Group A had the highest amount of statistically positive results along with Group R. Group A showed positive results in the parameters elevation, loss of lateral rotation in adduction, and medial rotation in abduction. Group R showed positive results in the parameters lateral rotation in adduction and loss of lateral rotation in adduction. On the other hand, Group R + C group was the one that presented the greatest number of statistically negative results, in the following parameters: elevation, lateral rotation in abduction, loss of lateral rotation in adduction, and medial rotation in abduction.

 Previous studies that evaluated ranges of shoulder motion after glenoid bone graft surgery demonstrated favorable results, with the vast majority of patients presenting ranges of motion similar to preoperative levels. ^11^ Lateral rotation is the main movement that presents alteration in the postoperative period of this type of surgery. ^38^^-^^40^

 Many patients have loss of lateral rotation after the Latarjet procedure. ^38^^-^^40^ Hovelius et al. ^38^ found an average loss of lateral rotation in adduction of 7.4 degrees and in abduction of 8 degrees. They discussed that this could be avoided with proper rehabilitation. 

 The Latarjet procedure is associated with loss of active range of motion, loss of active lateral rotation up to 19 degrees ^40^ , and minimal loss of active medial rotation. ^41^

 Ernstbrunner et al. ^40^ conducted a study in which they followed patients undergoing the Latarjet procedure as primary surgery for shoulder instability, and in the mean follow-up of 8.4 years there was only a loss of 4 degrees of lateral rotation compared to the contralateral side. Lafosse and Boyle ^42^ reported a loss of 18 degrees of lateral rotation and Sinha et al. ^39^ reported a loss of lateral rotation of 10 degrees and medial rotation of 6 degrees. 

It is worth mentioning that for most sports this lateral rotation deficit does not bring repercussions, but for pitching patients it can mean loss of performance and termination of the sports career. Thus, in this group of patients, the glenoid bone graft surgery should be chosen carefully.

An important aspect to be observed is that, although Group A presented statistically positive results in the evaluated parameters regarding lateral rotation, both in adduction and abduction, this group presented statistically negative results. Group R had the best results concerning lateral rotation, presenting statistically positive results regarding lateral rotation in adduction, lateral rotation in abduction, and loss of lateral rotation in adduction. Our hypothesis was that Group R + C group would present the best results regarding ranges of motion, but this did not occur. We believed that, by using clinical and radiological criteria, there would be a better selection of patients, but groups A and R presented more favorable results. The clinical criteria used may have little influence or are not the most relevant for surgical indication.

 In previous studies, glenoid bone graft surgery showed good functional results, despite a relatively high rate of complications. ^11^ The aim of our study was to determine which surgical indications are related to a better result regarding ranges of motion, since this surgery is often indicated in young patients and athletes, in whom the expectation of surgery is high. Our study seeks to help by suggesting the best forms of indication so that the best possible functional result is achievable. 

 The overall methodological quality of the studies was low. This is a factor that influenced the results of this systematic review. By the Downs & Black checklist ^13^ score, seven studies were classified as weak, three as regular, and two as good. 

Limitations of this systematic review: the parameters evaluated in the studies and types of surgeries were considerably variable. The techniques used by the surgeons in the studies and the indications in each subgroup were not the same in the selected studies. The other limitations of the study are inherent to those of systematic reviews. The overall sample of patients included patients of different ages, functional demands, numbers of dislocation episodes, time to surgery, making it challenging to apply the results to a particular patient. Nonetheless, our systematic review is the first to attempt to determine which surgical indications would lead to a better outcome regarding ranges of motion.

## CONCLUSION

In this systematic review, the subgroups presented variable results in the evaluated parameters; however, the groups of arthroscopic and radiological indications presented the highest amount of positive results, and the latter group presented the best results regarding lateral rotation.
